# Postural Influence on Ventilation Efficiency and Relationship With Oxygen‐Enhanced MRI in Cystic Fibrosis

**DOI:** 10.1002/ppul.71724

**Published:** 2026-07-04

**Authors:** Constantinos Efthyvoulou, Thomas Semple, Mary Abkir, Marta Tibiletti, Simon Padley, Geoff J. M. Parker, Jane C. Davies, Christopher Short

**Affiliations:** ^1^ National Heart and Lung Institute Imperial College London London UK; ^2^ Department of Paediatrics, Royal Brompton & Harefield Hospitals Part of Guys and St Thomas' Trust London UK; ^3^ Centre for Paediatrics and Child Health Imperial College London UK; ^4^ Lung Clearance Index Core Facility European Cystic Fibrosis Society London UK; ^5^ Bioxydyn Limited Manchester UK; ^6^ Department of Medical Physics & Biomedical Engineering, UCL Hawkes Institute University College London London UK

**Keywords:** cystic fibrosis, multiple breath washout, oxygen‐enhanced magnetic resonance imaging, postural position, supine, trapped air, under ventilated lung units

## Abstract

**Background:**

Pulmonary function tests (PFTs) and medical imaging in cystic fibrosis (CF) occasionally show discrepancies in severity levels, potentially due to different postures during testing (seated PFTs and supine imaging). To assess this, we utilised multiple breath washout with Short extension (MBW_ShX_) and oxygen‐enhanced MRI (OE‐MRI). MBW_ShX_ is a more sensitive PFT and offers novel insights into global ventilation efficiency by incorporating signal from under‐ventilated lung units (UVLU). We hypothesised there would be a significant difference in MBW_ShX_ parameters between seated and supine postures and aimed to compare the relationship between MBW_ShX_ obtained in both posture and OE‐MRI.

**Methods:**

Twenty three CF patients performed two seated and at least 1onesupine MBW, along with same day OE‐MRI. MBW_ShX_ outcome measures included conventional lung clearance index (LCI_2.5_), and two novel parameters: LCI_ShX_ and UVLU. The primary OE‐MRI parameter was the ventilation defect percentage (VDP%).

**Results:**

We demonstrated a significant increase in LCI_ShX_ (median difference [IQR]:2.00[0.16; 2.61]; *p* < 0.01) and UVLU (0.66[0.11; 2.22]; *p* < 0.05) from seated to supine posture, but no significant change in LCI_2.5_ (0.27 [−0.21; 0.89]; *p* = 0.06). Moreover, we showed strong correlation between MBW and VDP% of OE‐MRI parameters across postures (*ρ* > 0.8). However, no differences were detected between seated and supine MBW correlations with OE‐MRI (LCI_2.5_, *p* = 0.77; LCI_ShX_, *p* = 0.90; UVLU, *p* = 0.29).

**Conclusion:**

We found significant postural differences in MBW_ShX_ parameters, with reduced global ventilation efficiency (increased LCI_ShX_) and increased UVLU in supine posture. However, the relationship between MBW and OE‐MRI remained unaffected by posture. In conclusion, supine MBW may offer novel clinical insights but does not enhance sensitivity for comparisons with medical imaging.

## 1 Introduction

Monitoring respiratory disease requires a multifaceted approach, which commonly relies on a combination of pulmonary function testing (PFT) and imaging [1]. The most common and widely regarded “gold standard” PFT is spirometry, with its primary parameter being the forced expiratory volume in the 1st second (FEV_1_). However, spirometry has limited sensitivity particularly when respiratory disease is either heterogenous or located in the peripheral airways. This has led to the emergence of Multiple Breath Washout (MBW) in recent decades with notable success in monitoring cystic fibrosis (CF) lung disease. MBW has been used as the primary endpoint in several CF clinical trials [2, 3, 4] and is being adopted by many specialist centres for clinical monitoring. The primary metric obtained is the Lung Clearance Index (LCI), which is calculated once the tracer gas concentration is “washed out” to <2.5% of the starting concentration, and therefore is termed LCI_2.5_. LCI_2.5_ is a measure of ventilation efficiency, which is a composite metric, encompassing gas mixing efficiency, physiological dead‐space and ventilation inhomogeneity [5]. MBW is performed during relaxed tidal breathing, making it more suitable than forced techniques for paediatric patients.

However, LCI_2.5_ is not without limitations, as it reveals little regarding occluded lung regions commonly referred to as ‘trapped air’ [6]. The occlusion of these lung regions arises from mucus plugs or collapsed airways, and they are very common in respiratory diseases such as CF, primary ciliary dyskinesia, asthma etc. However, we consider the term “trapped air” to be something of a misnomer; several studies demonstrate that these areas are still ventilated albeit at a slower rate likely through collateral ventilation channels and pendelluft [7, 8]. To address this limitation, we developed a Short extension to MBW (MBW_ShX_), which enables access and quantification of signal from under‐ventilated lung units (UVLU) [9]. Two new parameters are generated, a global parameter termed LCI_ShX_ and a marker of the extent UVLU. MBW_ShX_ uses a slow vital capacity (SVC) after the conventional end of the washout, with the derived signal calculated into the same units as LCI_2.5_. We recently showed that the parameters from MBW_ShX_ had a very strong relationship with “trapped air” from spirometry‐controlled CT. We recently demonstrated that MBW_ShX_ parameters show a strong relationship with “trapped air” measured by spirometry‐controlled CT, with significantly improved sensitivity compared to spirometry, plethysmography, and previously described MBW‐derived indices of trapped air [9].

Despite the improved information content from MBW_ShX_, any PFT parameter will lack spatial information, leaving an important role for medical imaging. Traditionally, the “gold standard” for structural lung monitoring has been computed tomography (CT) [10]. However, its reliance on ionising radiation limits its frequency of use [11], particularly among paediatric patients and only provides a snapshot of function which limits its utility. These limitations have led to the emergence of lung MRI, which can provide both structural and functional information. To date, investigators have focused on using hyperpolarised (HP) gases such as ^3^helium or ^129^xenon to provide ventilation information and have demonstrated good correlations with CT [12] and LCI_2.5_ [13]. In parallel, several non‑hyperpolarised MRI techniques have been developed, including phase‑resolved functional lung (PREFUL) MRI [14] and related Fourier‑based approaches, which exploit signal variations during free breathing to derive ventilation information, as well as ^19^F MRI using inhaled fluorinated gases [15]. While these techniques have shown feasibility and sensitivity in cystic fibrosis and other lung diseases, HP gas MRI and ^19^F MRI require specialised hardware, dedicated gases, and complex logistical support, which limits their availability and routine clinical use. PREFUL MRI, although widely accessible, relies on indirect signal surrogates and is sensitive to motion, acquisition parameters, and post‐processing choices. We therefore focused on oxygen‐enhanced MRI (OE‐MRI), which uses molecular oxygen as an endogenous contrast agent, can be implemented on standard clinical MRI systems, and shows promise as a scalable functional lung imaging technique suitable for broader clinical translation. It relies on the paramagnetic properties of molecular oxygen (O_2_), allowing it to be used as a contrast agent. Therefore, the signal obtained can be exploited for functional medical imaging [16]. A range of OE‐MRI measurements have been proposed in the literature [17, 18, 19]. Here we focus on ventilation defect percentage (VDP%‐ absence of ventilation, similar to VDP% derived from hyperpolarised‐gas MRI), and ∆R_2_*, a ventilation signal score derived from a T_2_* sequence to capitalise on changes in magnetic susceptibility gradients caused by elevated alveolar oxygen levels. This technique potentially offers a more direct assessment of ventilation and is used here. We recently demonstrated the feasibility, reproducibility, and sensitivity of this approach in people with (pw)CF, including its ability to detect longitudinal progression of lung disease, supporting its potential role as a meaningful functional imaging biomarker [20].

PFTs and medical imaging provide valuable, complementary insights for detecting and monitoring lung disease. However, medical imaging is typically performed in the supine posture, while PFTs are usually performed upright; hence, physiological lung alterations between the two postures need to be considered. Studies using dual‐position CT scans and MBW have shown that transitioning from a seated to supine posture elicits changes in lung volumes [21, 22, 23]. While a reduction in total lung capacity is not always reported, it is widely acknowledged that there is a pronounced reduction in functional residual capacity (FRC) when moving from upright to supine [24]. Specifically, FRC reduction originates from the gravitational shifting of abdominal organs and mediastinal contents when supine, partially impeding diaphragmatic recoil [25]. These physiological differences in postures may be the reason for weaker than expected correlations between LCI and imaging outcome. Particularly as FRC is the denominator in the equation to calculate LCI (Cumulative expired volume/FRC).

There is limited research on the effect of posture on LCI, and previous studies conflict on the differences between supine and seated LCI and the relationship with imaging. Smith et al. and Ramsey et al. demonstrated that LCI_2.5_ increases (worsens) in the supine compared to the seated posture in both CF patients and healthy controls (HCs) [22, 26]. Conversely, Gustafsson demonstrated no significant difference in LCI in asthmatic children between the 2 postures, but an increase in the volume of trapped air while supine [24]. Ramsey et al. also reported a stronger correlation between CT and LCI_2.5_ when the latter was measured supine rather than seated [26]. In contrast, similar studies by Marshall et al. and Smith et al. demonstrated a greater correlation of HP‐MRI with seated LCI_2.5_ than supine LCI_2.5_ [22, 27]. However, no studies have looked at the effect of posture on UVLU outcomes.

### 1.1 Hypothesis and Aims

We hypothesise that there is a significant difference between MBW_ShX_ parameters (LCI_2.5_, LCI_ShX_ and UVLU) obtained whilst seated and in supine posture. The aims of our study are to determine whether there is a significant difference in postural position on MBW_ShX_ parameters and to understand the impact of this on their relationship with OE‐MRI parameters.

## 2 Methods

### 2.1 Multiple Breath Washout With Short Extension (MBW_ShX_)

A cross‐sectional study was carried out in which MBW and OE‐MRI were performed on the same day. PwCF 6 years of age and older were recruited, from an ongoing, ethically approved study (IRAS‐294686) at the Royal Brompton Hospital. Written consent was obtained from all participants aged 16 and above. For participants under 16, written consent was obtained from parents/guardians, along with written assent from the child. Exclusion criteria included standard contraindications to MRI [28].

#### 2.1.1 Multiple Breath Washout With Short Extension (MBW_ShX_)

MBW_ShX_ was performed using the Exhalyzer D (Ecomedics AG, Switzerland, Spiroware software version 3.3.1) in accordance with the ERS/ATS consensus statement [29]. MBW files were analysed according to central over‐reading centre standards, by a qualified over‐reader [30]. For MBW_ShX,_ participants performed a slow vital capacity (SVC) after three consecutive breaths had been obtained under the target N_2_ concentration (2.5% of the starting concentration); the addition of MBW_ShX_ requires ~2 min extra in the testing session (three MBW runs) [9]. In brief, the signal and derived parameters are calculated from changes in N_2_% with the SVC breath compared to breath immediately prior and standardised by the patients FRC. If the N_2_% remains above the target concentration after the SVC, tidal breathing is continued until it has fallen back under target concentration for three consecutive breaths. Each MBW test comprised at least two technically acceptable seated trials and at least 1 technically acceptable supine trial [29]. Mean LCI_2.5_ values were used, whereas LCI_ShX_ and UVLU values were derived from the trial with the largest change in N_2_ concentration brought on by the SVC breath, as its effort dependent manoeuvre. The order of posture for the first MBW trial (seated or supine) was determined using a random number generator. Quantification of UVLU was as previously described [9, 20, 31], but in brief, represents a functional representation of the amount of under‐ventilated lung units that are accessed and released during the SVC manoeuvre, reflecting slowly communicating regions of the lung. Further details can be found in the supplementary material.

### 2.2 Oxygen‐Enhanced Magnetic Resonance Imaging (OE‐MRI)

Images were acquired using an IV contrast‐ and sedation‐free protocol on a 1.5 T MR scanner (Siemens Aera) during *a* < 22 min protocol. Dynamic OE‐MRI was acquired using a multi‐slice Double Echo FLASH acquisition (TEs = 0.98/2 ms; TR = 16 ms; flip angle = 5°), with 360 repetitions and a temporal resolution of 1.5 s. Five 10 mm thick coronal images were acquired during free tidal breathing, with a field of view of 450 × 450 mm^2^ and an in‐plane spatial resolution of 4.7 × 4.7 mm^2^. Images were acquired over a 9‐min period alternating from medical air (120 s) to 100% oxygen (210 s) and back to medical air (210 s) [19]. Gases were delivered via oxygen blender (15 L/min, Low Flow Blender; Inspiration Healthcare, Leicester, UK) with subjects free breathing through a non‐rebreathe mask (Intersurgical, ECOLite^TM^). This oxygen signal is derived via the change in the R_2_*(ΔR_2_* = 1/∆T_2_*) signal during the gas delivery protocol. The primary parameter (via ΔR_2_*) is the ventilation defect percentage (VDP%), which represents the volume of lungs where ventilation is extremely limited or not present [32]. Further details for MRI analysis and methodology are provided in the supplementary material.

### 2.3 Statistical Analysis

Statistical analysis was performed using Prism 10 (GraphPad Software Inc, San Diego, CA). An *a priori* power calculation based on the primary within‐subject hypothesis indicated that a sample size of 16 participants would provide 80% power, and a sample size of 20 participants would provide approximately 90% power, to detect a median difference of 0.8 using a Wilcoxon signed‐rank test.

The distribution of data was assessed for normality using the Shapiro‐Wilk test. The Wilcoxon signed rank test was used to assess differences between seated and supine MBW testing. Data are presented as median values and interquartile ranges. The relationship between pulmonary function parameters and OE‐MRI was determined using Spearman's rank‐order correlation. Fisher *Z*‐Transformation test was used to compare the correlation coefficients between seated and supine posture with OE‐MRI (MedCalc Software Ltd, Ostend, Belgium). *p* < 0.05 was considered statistically significant.

## 3 Results

### 3.1 Cohort Demographics and Baseline Characteristics

Baseline characteristics are presented in Table 1. The study included 23 pwCF with median age of 14.4 years. All patients completed MBW_ShX_ and spirometry whilst OE‐MRI data is available for 22 of the 23 participants, as one declined to take part in MRI.

**Table 1:** Cohort demographics and baseline characteristics.

Number of participants	23
Male%	61%
**Medians (IQR)**	
Age (years)	14.4 (12.1; 27.3)
Height (cm)	162 (149; 177)
Weight (kg)	55.0 (39.5; 65.0)
ppFEV1	87.0 (74.0; 96.0)
ppFVC	97.0 (86.0; 106.0)
VDP% (*n* = 22)	11.8 (5.96; 20.3)
ΔR_2_* (*n* = 22)	0.067 (0.054; 0.082)
LCI_2.5_	7.20 (6.79; 11.06)
LCI_2.5_ Supine	7.35 (6.72; 12.3)
LCI_ShX_	11.7 (7.44; 21.1)
LCI_ShX_ Supine	14.0 (7.91; 23.1)
UVLU	3.72 (0.49; 9.78)
UVLU supine	6.25 (0.58; 9.97)
FRC	1.70 (1.42; 2.23)
FRC Supine	1.38 (1.13; 2.07)
CEV (L)	14.5 (9.54; 23.4)
CEV Supine (L)	10.8 (7.40; 22.2)
Vt (mL)	466 (416; 621)
Vt Supine (mL)	462 (413; 623)

Abbreviations: ΔR2*, Change in R2*; CEV, Cumulative Expired Volume; FRC, Functional Residual Capacity; LCI2.5, Lung Clearance Index at 2.5%; LCIShX, Lung Clearance Index with Short extension; ppFEV1, percent predicted Forced Expiratory Volume in 1 s; ppFVC, percent predicted Forced Expiratory Volume; VDP%, Ventilation Defect Percentage; Vt, Tidal volume; UVLU, Under Ventilated Lung Units.

### 3.2 The Effect of Postural Position on MBW_ShX_ Parameters

**Figure 1:**
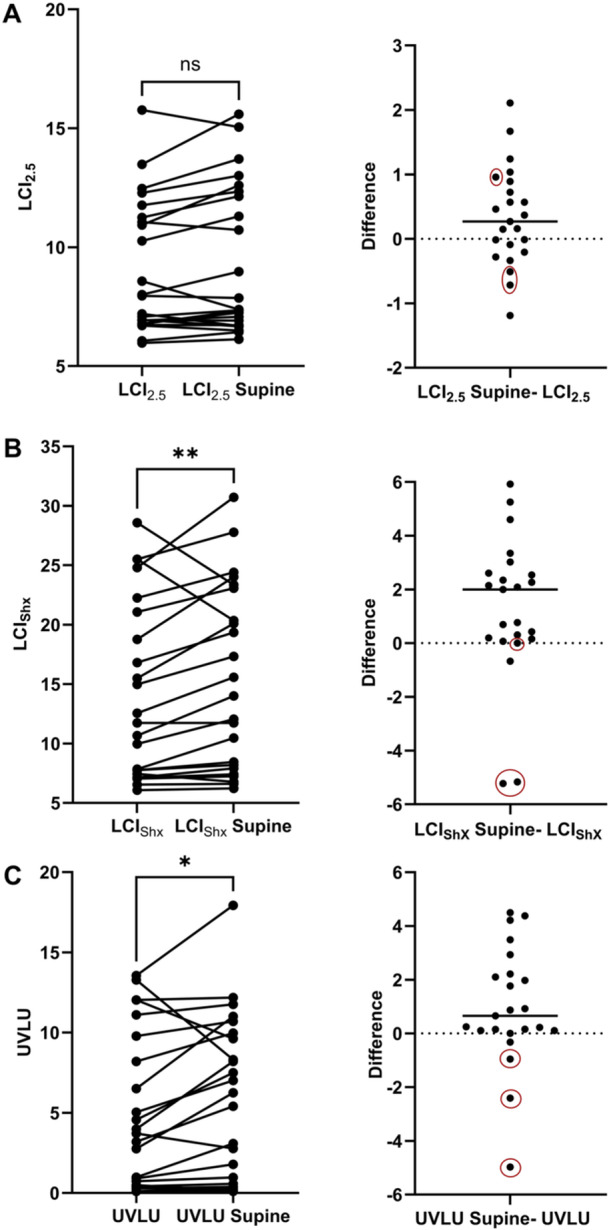
Changes in MBW Ventilation Efficiency parameters from seated to supine posture in all participants (*n* = 23). Graphs on the left‐hand side represent matched individual participant values between the 2 postures. Difference plots on the right‐hand side present the difference (supine—seated) between the 2 positions. Horizontal lines in difference plots represent median change from seated to supine. Participants with discordant postural responses are indicated by red circles. (A) Postural effect on LCI_2.5_ (median change [IQR]; (0.27 [−0.21; 0.89]; *p* = 0.060)). (B) Postural effect on LCI_shx_ (2.00 [0.16; 2.61]; *p* = 0.0066). (C) Postural effect on extent of UVLU (0.66 [0.11; 2.22]; *p* = 0.021). Statistical testing for all 3 parameters was performed using the Wilcoxon signed ranked test. *p* < 0.05 was considered statistically significant *p < 0.05; **p < 0.01, CF, Cystic Fibrosis; HCs, Healthy Controls; IQR, Interquartile Range; LCI_2.5_, Lung Clearance Index at 2.5%; LCI_ShX_, Lung Clearance Index with Short extension; ns, not significant; UVLU, Under Ventilated Lung Units. [Color figure can be viewed at wileyonlinelibrary.com]

The postural effect on MBW parameters is demonstrated in Figure 1. LCI_2.5_ showed no significant difference between seated and supine positions (median difference [IQR]: 0.27[−0.21;0.89]; *p* = 0.06) (Figure 1A). However, both LCI_ShX_ (2.00[0.16;2.61]; *p* = 0.0066) and UVLU (0.66 [0.11; 2.22]; *p* = 0.021) were significantly greater in the supine than seated posture (Figure 1B,C). When expressed as percentage change, the median increase was 3.8% (IQR − 3.0; 8.5) for LCI_2.5_ and 9.7% (2.6; 23.9) for LCI_ShX_. No significant relationship was observed between baseline MBW_ShX_ parameters and percentage change in any MBW outcome.

### 3.3 Seated Versus Supine Posture Effect on Lung Volumes

**Figure 2:**
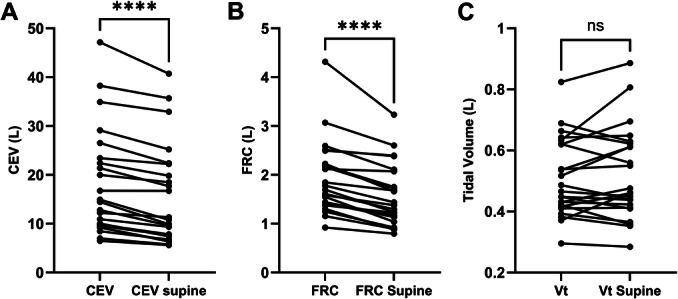
Changes in MBW Volume Parameters from seated to supine posture (*n* = 23). Statistical testing was performed using the Wilcoxon signed ranked test. *p* < 0.05 was considered statistically significant. (A) Postural effect on CEV (Median difference [IQR]: −2.57 L [−3.70; −1.50 L]; *p* < 0.0001). (B) Postural effect on FRC (−0.31 L [−0.46; −0.17 L]; *p* < 0.0001). (C) Postural effect on Vt (−8.40 mL [−38;62 mL]; *p* = 0.62). ****p < 0.0001. CEV, Cumulative Expired Volume; CF, Cystic Fibrosis; FRC, Functional Residual Capacity; HCs, Healthy Controls; IQR, Interquartile Range; ns, not significant; Vt, Tidal volume.

The effect of posture on MBW volumes is shown in Figure 2. A significant reduction in cumulative expired volume (CEV) was observed between seated and supine posture (−2.57 L[−3.70;‐1.50 L]; *p* < 0.0001) (Figure 2A). Similarly, FRC also was significantly lower in the supine posture (−0.31 L[−0.46; −0.17 L]; *p* < 0.0001) with median change of 19.5% (Figure 2B). A strong and statistically significant correlation was found between the change in CEV and the change in FRC (*ρ*: 0.61; *p* < 0.0022). Furthermore, tidal volume was similar in the two postures (−8.40 mL [−38; 62 mL]; *p* = 0.62) (Figure 2C).

### 3.4 Participants With Discordant Postural Responses

Although UVLU was significantly greater in the supine position, three participants showed a large reduction in UVLU, which exceeded the expected variability between tests. Furthermore, two of these three participants also demonstrated a reduced LCI_ShX_ while supine, alongside relatively smaller changes in FRC between postures (2% and 9%, median 19.5%). In these same participants, LCI_2.5_ showed heterogeneous behaviour between postures, with one individual demonstrating a large relative worsening (~12%) and the remaining participants demonstrating small improvements (3.6% and 4.4% respectively), which are within the expected variability of the measure. The distribution of disease in these three participants differs from the rest of the cohort as they had predominant upper lobe disease with relatively spared lower lobes. This upper‑lobe predominant pattern may partly explain their paradoxical improvement in UVLU while supine.

### 3.5 Relationship of MBW Parameters With OE‐MRI

**Figure 3:**
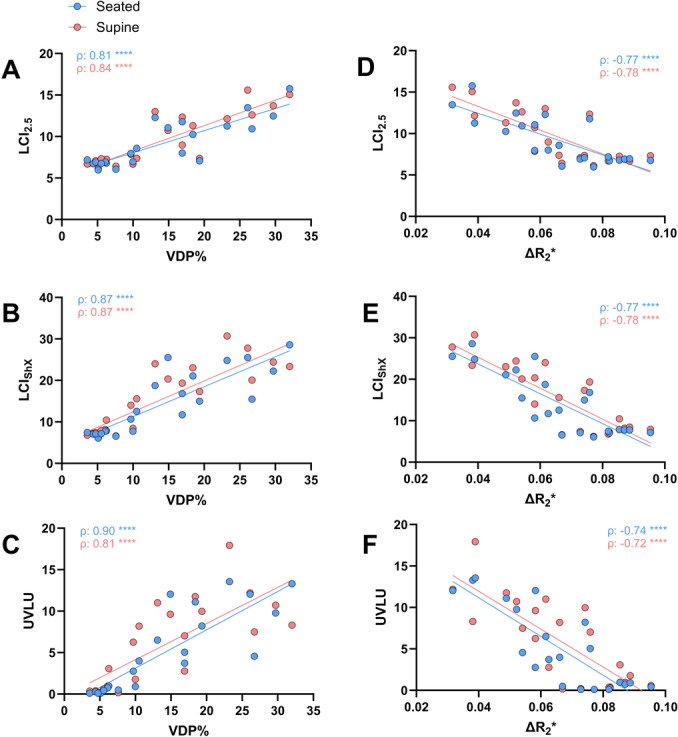
Correlations between MBW Ventilation Efficiency parameters and VDP% from OE‐MRI (*n* = 22). All 3 MBW parameters (LCI_2.5_, LCI_ShX_ and UVLU) correlated strongly and significantly with VDP% and ΔR_2_* derived from OE‐MRI. Blue circles represent seated participants and red circle represent supine participants. Blue and Red Lines represent the line of best fit for seated and supine posture respectively, to serve as visual aid in interpretation. *p* < 0.05 was considered statistically significant. (A) Correlation of postural LCI_2.5_ and VDP%. (B) Correlation of postural LCI_ShX_ and VDP%. (C) Correlation of postural UVLU and VDP%. (D) Correlation of postural LCI_2.5_ and ΔR_2_*. (E) Correlation of postural LCI_ShX_ and ΔR_2_*. (F) Correlation of postural UVLU and ΔR_2_*. ****p < 0.0001. LCI_2.5_, Lung Clearance Index at 2.5%; LCI_shx_, Lung Clearance Index with Short extension; UVLU, Under Ventilated Lung Units; VDP, Ventilation Defect Percentage. [Color figure can be viewed at wileyonlinelibrary.com]

The relationships between MBW parameters in the two different postures and OE‐MRI parameters, VDP% and ΔR_2_*, are presented in Figure 3. VDP% demonstrated strong and significant correlations with all MBW parameters in both postures (LCI_2.5_ seated *ρ*: 0.81, *p* < 0.0001; LCI_ShX_ seated *ρ*: 0.86, *p* < 0.0001; UVLU seated *ρ*: 0.90, *p* < 0.0001; LCI_2.5_ supine *ρ*: 0.84, *p* < 0.0001; LCI_ShX_ supine *ρ*: 0.87, *p* < 0.0001; UVLU supine *ρ*: 0.81, *p* < 0.0001). No statistically significantly differences in Spearman's *ρ* values between postures were observed (LCI_2.5_, *p* = 0.77; LCI_ShX_, *p* = 0.90; UVLU, *p* = 0.29).

Similarly, ΔR_2_* exhibited strong and significant correlations with MBW in both postures (LCI_2.5_ seated *ρ*: 0.77, *p* < 0.0001; LCI_ShX_ seated *ρ*: 0.77, *p* < 0.0001; UVLU seated *ρ*: 0.77, *p* < 0.0001; LCI_2.5_ supine *ρ*: 0.78, *p* < 0.0001; LCI_ShX_ supine *ρ*: 0.78, *p* < 0.0001; UVLU supine *ρ*: 0.72, *p* < 0.0001). Again, no statistically significant differences in correlation were observed between seated and supine postures (LCI_2.5_, *p* = 0.94; LCI_ShX_, *p* = 0.94; UVLU, *p* = 0.73).

## 4 Discussion

In this study, we assessed the impact of postural positioning on MBW_ShX_ outcomes and their corresponding correlations with OE‐MRI. Both methods were well tolerated, with a 100% success rate; however, one patient declined to take part in the MRI portion of the study due to claustrophobia. Whilst the sample size was limited, it was broadly representative of the UK CF population; ppFEV_1_ 43%–111% and age 6–57 years. We found that postural position significantly impacts the novel parameters from MBW_ShX_ (LCI_ShX_ and UVLU), with significantly higher values in the supine posture. We also found the supine position caused a significant reduction in MBW lung volumes (FRC and CEV). In contrast, conventional LCI_2.5_ exhibited bidirectional and unpredictable variability between the two positions, with no statistically significant difference. Despite these posture related differences, we found no significant difference in the strength of association between MBW parameters obtained in the two postures and OE‐MRI.

### 4.1 Interpretation of Results

Our findings reveal a no significant group change in LCI_2.5_ between seated and supine posture. Although the overall pattern of LCI_2.5_ may suggest a slight increasing trend (median difference of 0.27), most changes fell within the expected physiological and technical variability of LCI_2.5_ (~10%–15%). Only five of the 23 participants exhibited a change greater than 10%, between the two postures, which may be considered clinically relevant [33]. Previous literature is conflicting whether postural position significantly increases LCI_2.5_. Studies by Ramsey and Smith et al. both reported a significant increase of LCI_2.5_ in the supine posture [22, 26], but notably both studies recruited exclusively paediatric patients, who typically have mild disease. Moreover, there are important methodological differences between our study and the study by Smith et al, which may account for the discrepancies [22]. The authors performed MBW using wash‐in of SF_6_ gas, whereas we used MBW using 100% O_2_ to washout resident N_2_. It is widely acknowledged that the LCI_2.5_ obtained from different gases and devices cannot be directly compared [5], due to differing software algorithms and diffusion properties. Differences between healthy lungs are small [34] but can be larger when assessing lungs with poorly ventilated lung regions. This is due to the rate of diffusion being inversely proportional to the size of the molecules. For instance, the particle size of N_2_ is 0.363 nm whereas SF_6_ is 290 nm which may explain how the derived values of SF_6_ are often lower than N_2_ MBW values [35]. Nevertheless, Ramsey et al. also utilised MBWN_2_ and still reported a significant increase in LCI_2.5_ in the supine posture [26]. Conversely, Gustafsson using MBWN_2_ in asthmatic children, found no significant postural effect on LCI_2.5_, emphasising the complexity of the influence of posture on MBW parameters [24].

Both LCI_ShX_ and the extent of UVLU increased significantly in the supine posture. Specifically, 13 of the 23 participants demonstrated a change in LCI_ShX_ greater than 10%, which may be considered clinically relevant [9]. These findings are consistent with the work of Gustafsson, who reported an increase in supine'volume of trapped air' in asthmatic patients using an alternative protocol and calculation method [24]. “Trapped air”/UVLU is an important pathophysiological feature in CF, and on CT it has been shown to be the best predictor of disease progression [10]. Furthermore, given the clinical practice of transitioning MBW testing from the supine to the seated position between infancy and preschool age, the observed changes in LCI_ShX_ and UVLU warrant attention. Whilst we do not dispute the impact of deadspace and testing differences, the interplay between communicating and non‐communicating lung units may be partially behind the improvement found in LCI_2.5_ when transitioning from infant to preschool testing [29, 35].

We found no statistically significant differences in relationships between OE‐MRI and any MBW parameters assessed in the seated and supine positions. This suggests that there is no additional value in performing MBW_ShX_ in the supine posture. However, the largest changes appeared in those with most marked disease and therefore this relationship should be explored further. We observed a strong correlation between VDP% derived from OE‐MRI and MBW_ShX_. The strength of these associations is comparable to those reported using hyperpolarised gas‐MRI (HP‐MRI) by Marshall et al. and Smith et al. [13, 27]. Interestingly, all MRI methods appear to have a much stronger relationship with MBW than does CT [26]. This is likely related to the additional functional data that is available with MRI whereas CT only provides a snapshot into this pathophysiology. In the case for OE‐MRI, the strong correlation may be attributed to the tidal breathing protocol of 100% Oxygen, which closely mirrors the MBW_ShX_ methodology [20]. The duration of the OE‐MRI protocol may facilitate more comprehensive ventilation of UVLU, likely via collateral ventilatory channels, which aligns more closely with the subject's own respiratory physiology, thereby reducing VDP% [7, 31].

### 4.2 Theoretical Considerations

FRC occurs at the point where the inward elastic recoil of the lungs and the outward recoil of the chest wall are balanced [5, 35]. In the supine posture, gravitational effects increase the overall lung elastic recoil, leading to a reduction in FRC [36]. Our findings support this physiological principle, demonstrating a 19.5% reduction in FRC in the supine position, which is in line with previously reported values [24, 34]. Given that both FRC and CEV exhibited a substantial reduction in the supine compared to the seated posture, we postulate that this reflects a smaller communicating lung volume being ventilated (‘washed out') during the test. This may be a consequence of increased lung resistance in the supine posture leading to greater airway closure or collapse compared to seated and potentially contributing to an increase in UVLU [37]. In pwCF with more severe disease who have bronchiectasis, inflammation or airway narrowing (bronchial wall thickening), this collapse appears to be exacerbated, further increasing UVLU [38].

A potentially important source of bias in LCI_2.5_ calculation is variation in tidal volume between MBW tests. Previous studies demonstrated that shallower breathing tends to overestimate LCI_2.5_, whilst deeper breathing underestimates it [39]. To minimise this source of bias, participants were instructed to maintain stable breathing volumes whenever possible, aided by breath‐to‐breath monitoring. Consequently, we found no significant difference in tidal volume between the postures and therefore mitigated its potential role in revealing significant differences in MBW_ShX_ parameters. Another possible contributor to postural differences in LCI_2.5_ may be the slow ventilation of UVLU through collateral channels, the extent of which varies between individuals [7]. It is possible that participants with increased LCI_2.5_ in the supine posture utilise collateral ventilation more than those who do not see an increase. In cases of severe disease, a single MBW trial can exceed 6 min in duration, potentially allowing time to partially “wash out” some of the UVLU. As a result, these UVLU may contribute a small amount to the LCI_2.5_ signal, thereby prolonging the test and leading to elevated LCI_2.5_ which is better aligned with their severity of disease. Although individuals with more advanced structural lung disease may be more susceptible to airway closure in the supine posture, we did not observe a direct association between baseline MBW severity and the magnitude of postural change, suggesting that these effects are not solely driven by disease severity.

**Figure 4:**
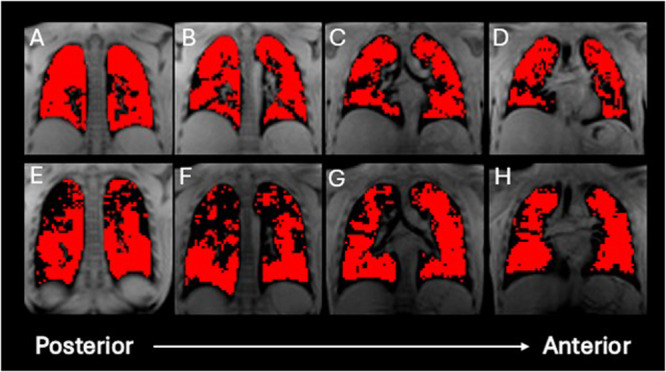
*OE‐*MRI maps showing ventilated voxels in red and ventilation defect in black. Four slices are positioned from posterior (left) to Anterior (right) for two patients with CF. Patient 1 —slices A–D, showing relatively spared posterior and upper lobes with patchy disease in the anterior slices. Patient 2—(E–H), majority of disease (ventilation defects) are in the posterior and upper lobes with the anterior slices demonstrating less disease. Patient 1 followed the consistent pattern of worsening extent of UVLU from MBW_ShX_ in the supine position compared to the seated position. Patient 2 however, was 1 out of 3 outliers with an improvement in UVLU in the supine position, which we hypothesise may be due to the predominant upper lobe and posterior disease deposition, which enjoys greater ventilation in the supine position compared to seating. CF, Cystic Fibrosis; MBW_ShX_, Multiple Breath Washout with Short extension; UVLU, Under Ventilated Lung Units. [Color figure can be viewed at wileyonlinelibrary.com]

Despite the overall group increase in UVLU in the supine posture, 3 participants exhibited a reduction in UVLU. 2 of these 3 participants also demonstrated reduced LCI_ShX_ alongside relatively smaller changes in FRC between postures (2% and 9% ‐ median 19.5%). This smaller alteration in FRC therefore resulted in less of a difference in the accessible and communication lung compared to the rest of the CF cohort. Additionally, all three participants had predominant upper lobe disease with relatively spared lower lobes, as observed on OE‐MRI reports (Figure 4). Previous research has shown that ventilation of the upper lobes increases in the supine compared to seated posture [40]. We therefore hypothesise that in these patients, UVLU in the upper lobes were more effectively ventilated and consequently “washed‐out” during the standard supine MBW test than in the seated. As a result, the relative UVLU [N_2_] detected was lower in the supine MBW than in the seated. Future studies specifically powered to assess regional ventilation changes across postures are warranted.

### 4.3 Limitations

This study is not without limitations. First this was a single‐centre study with a modest sample size, which may limit generalisability and statistical power for subgroup analysis. While the study was adequately powered for its primary within‐subject comparison, larger multi‐centre studies are required to confirm these findings across broader CF populations. OE‐MRI remains a developing technique and is not yet fully standardised for routine clinical use. In this study, we employed a T_2_*‐based acquisition to capitalise on oxygen‐induced changes in magnetic susceptibility gradients caused by elevated alveolar oxygen concentration, providing a physiologically meaningful functional signal closely related to regional ventilation. As with all R_2_ based measures, ΔR_2_* may also be influenced by lung tissue density and, in principle, lung volume. However, under tidal breathing conditions, the use of 100% oxygen is not associated with functionally relevant changes in lung inflation in children or adults, a finding supported by extensive MBW literature using nitrogen washout. All acquisitions were performed with identical protocols and within‐session normoxic baselines, minimising physiological and inter‐subject variability. A fixed oxygen inhalation duration was applied across participants to ensure consistency, although this may not be optimal for all individuals, particularly those with severe disease or slower ventilation kinetics. In addition, a multi‐slice 2D OE‐MRI protocol was used, which limits full spatial lung coverage and may underestimate focal ventilation defects; future 3D acquisitions would address this at the expense of reduced signal to noise and longer acquisition times. Finally, OE‐MRI currently lacks inter‐site standardisation, normative reference ranges, and consensus processing pipelines, limiting direct comparison across studies but not the internal validity of the posture‐dependent comparisons presented here. Furthermore, in a small number of participants with more advanced lung disease, only one technically acceptable supine MBW trial could be obtained due to time constraints, which may have reduced within‐subject precision in those cases.

## 5 Conclusion

The results of our study demonstrate a significant difference in MBW_ShX_ parameters between seated and supine posture. Specifically, both LCI_ShX_ and extent of UVLU showed significant increase in the supine position. However, LCI_2.5_ showed no significant group difference. To our knowledge, the observed increase of UVLU in the supine position is novel in CF cohorts and may aid in interpreting differences between disease severity derived from imaging and MBW parameters. Additionally, we found no significant difference in the strength of correlations between different MBW and OE‐MRI parameters across postures. Therefore, our study suggests that performing MBW in the supine posture does not offer additional comparability in relation to medical imaging.

## Author Contributions

**Constantinos Efthyvoulou:** data curation, writing – original draft, writing – review and editing, project administration, formal analysis, investigation. **Thomas Semple:** writing – review and editing, supervision, funding acquisition, resources. **Mary Abkir:** data curation, project administration, writing – review and editing. **Marta Tibiletti:** formal analysis, writing – review and editing, software. **Simon Padley:** supervision, writing – review and editing, resources. **Geoff J. M. Parker:** writing – review and editing, formal analysis, funding acquisition, software. **Jane C. Davies:** supervision, writing – review and editing, funding acquisition, conceptualization, resources. **Christopher Short:** conceptualization, writing – review and editing, writing – original draft, supervision, formal analysis, methodology, project administration, investigation.

## Conflicts of Interest

The authors declare no conflicts of interest.

## Supporting information

Supporting File

## Data Availability

The data that support the findings of this study are available on request from the corresponding author. The data are not publicly available due to privacy or ethical restrictions.
