# Supplementary material for: Postural Influence on Ventilation Efficiency and Relationship With Oxygen‐Enhanced MRI in Cystic Fibrosis
Source: Pediatr Pulmonol. 2026 Jul 4;61(7):e71724. doi: 10.1002/ppul.71724 (PMC13332698; doi:10.1002/ppul.71724)
Supplement: Supplementary file 1 — Supporting File [file PPUL-61-0-s001.docx]

**Supplementary material**

**Postural Influence on Ventilation Efficiency and Relationship with Oxygen-Enhanced MRI in Cystic Fibrosis**

**Constantinos Efthyvoulou^1^, Thomas Semple ^1,2,3^, Mary Abkir ^1,2,4^ , Marta Tibiletti^5^ _,_ Simon Padley^1,2^, Geoff JM Parker ^5,6^, Jane C Davies ^1,2,3,4^, Christopher Short^1,2,4*^**

1 National Heart and Lung Institute, Imperial College London, London, United Kingdom

2 Royal Brompton & Harefield Hospitals, part of Guys and St Thomas’ Trust, Department of Paediatrics, London, United Kingdom

3 Centre for Paediatrics and Child Health, Imperial College London, UK

4 Lung Clearance Index Core Facility, European Cystic Fibrosis Society, London, United Kingdom

5 Bioxydyn Limited, Manchester, UK

6 UCL Hawkes Institute, Department of Medical Physics & Biomedical Engineering, University College London, London, UK

**Corresponding author:**

Christopher Short, PhD, MRes, BSc

National Heart and Lung Institute

Imperial College London

Emmanuel Kaye Building, 1b Manresa Road, London, SW3 6LR

[christopher.short@imperial.ac.uk](mailto:christopher.short@imperial.ac.uk)

**Methods**

**Oxygen enhanced-MRI analysis**

Data analysis was run using Bioxydyn proprietary image analysis software, VoxelFlow. Where manual intervention was required (e.g., for segmentation), this was performed by a single member of the Bioxydyn staff. In brief, the main analysis steps are:

- **Segmentation:** left and right lung masks were created on a reference expiratory image using an in-house masking tool.
- **Registration:** First echo images were registered via a deformable registration to a reference expiratory image using the ANTs library [1]. The second echo images were then registered applying the same transformation.
- **Extract respiratory signal:** The change in lung volume over time (R(t)) was extracted by applying each transformation to the mask and extracting the area of the deformed segmentations. Outlier rejections was applied based on R(t), excluding timepoint where the z-score was higher than 2, indicating episodes of deep breathing or coughing.
- **∆R_2_*:** ∆R_2_* is calculated from the two images acquired at each time point, using the formula (log(S(TE_1) - log(S(TE_2))/ (TE_2 – TE_1), where S(TE_1) is the MR signal at the shortest TE and S(TE_2) is the MR signal at the highest TE.
- **Calculate Oxygen enhancement map:** oxygen enhancement is calculated as the difference between a ‘Oxygen’ R_2_* image and a ‘baseline’ R_2_* image. The ‘Oxygen’ image is the R_2_* average over the second part of the oxygen inhalation period, the ‘baseline’ image is the R_2_* average over timepoint 10 to the start of oxygen inhalation. The exclusion of the first 10 timepoints is necessary due to possible MR artefacts that may be present in such acquisitions if the MR signal has not reached a plateau.
- **Calculate OE-VDP%:** ventilation defect percentage (VDP%) was calculated directly from the Oxygen enhancement map using a histogram-based method described by Obert et al [2] to discriminate which voxels are to be considered ventilated and which voxels are not considered to have received sufficient oxygen during the length of the oxygen inhalation. VDP% is the percentage of unventilated voxels within the lung masks.

**Multiple breath washout with Short extension (MBW_ShX_)**

The usual MBW end of test would occur once the subject had produced 3 consecutive tidal breaths under the target concentration (1/40^th^) of the starting [N_2_]. For MBW_ShX_, five such breaths were required following which the subject was instructed to take a slow maximal inspiration followed by a slow expiration to residual volume (often termed a slow vital capacity (SVC)). The subject was then instructed to take a tidal inspiration and return to tidal breathing. The test concluded after the end-tidal [N_2_] was below 1/40^th^ of the starting [N_2_] for the second time for three consecutive tidal breaths (Figure 8).


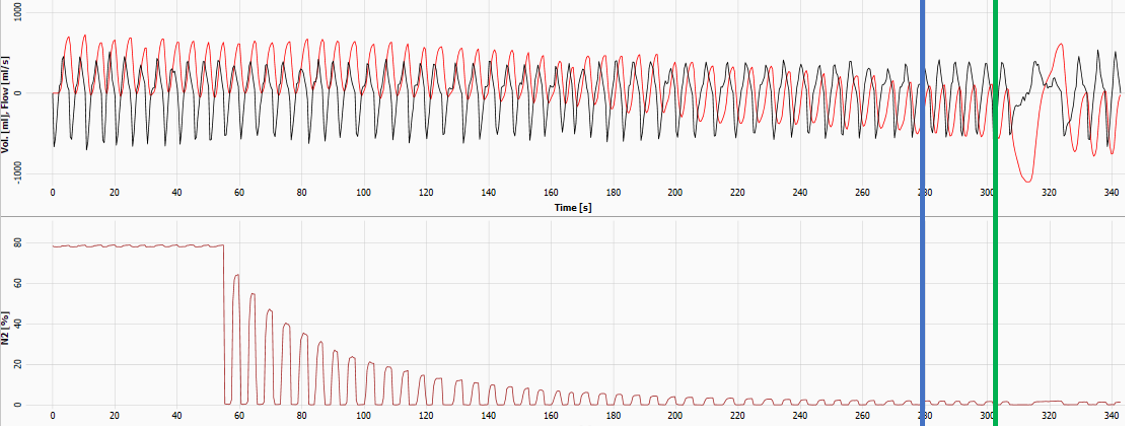


*Figure S1- Screen shot of MBW with Short extension. Top red line is volume, top black line is flow and nitrogen is the burgundy line. Slow vital capacity (SVC) performed after conventional MBW would be stopped. Blue line represents the target breath (first of three tidal breaths <2.5% of staring concentration). Green line represents where conventional MBW would stop.*

The calculation of UVLU follows the physiological concepts derived from slope analysis with the change in [N_2_] serving as a proxy and moment ratios with a similar area under the curve analysis.

The variables below are taken directly from the commercial software and all cumulative expired volume (CEV) and functional residual capacity (FRC) calculations are corrected for equipment dead-space.

The conventional method was used to calculate the LCI_2.5_: CEV / FRC

Quantification of UVLU was based on a two-step calculation requiring the [N_2_], FRC and CEV obtained from both the immediate pre-manoeuvre breath (PB) and the manoeuvre breath (MB).

Step 1- The change in Nitrogen concentration [N_2_], between the MB and the PB, multiplied by the volume (L) of the expiration of the MB. This is divided by the change in FRC (FRC_MB_) in addition to the subject’s FRC at LCI_2.5_. This part of the calculation is to ensure it is tailored to the individual’s lung volume as well as the size of the MB. Part 2- If there were substantial UVLU the breath(s) after the MB would have a higher [N_2_] than the original target concentration. In such cases the additional volume from these breaths that have a greater [N_2_] than the target [N_2_]; this is termed sustained CEV (sCEV). Again, the extra volume from these breaths was divided by any changes in FRC (sFRC).

To try to ensure that the subject did expire to RV; there was verbal encouragement, the manoeuvre was assessed, and the volume was cross-checked against spirometry readings. With this Short extension to MBW methodology we propose two new variables. Firstly, a functional representation of the amount of under-ventilated lung units that are released by the MB which we termed UVLU. In addition, we established a new marker of global lung health termed LCI_ShX_.

*UVLU = [(ΔN_2_CET_Norm_ * ΔCEV/ Δ FRC_MB_ + FRC) + (Δ sCEV/ ΔsFRC)]*

*LCI_ShX_ = [(ΔN_2_CET_Norm_ * ΔCEV/ Δ FRC_MB_ + FRC) + (Δ sCEV + CEV/ ΔsFRC + FRC)]*

These variables are unitless and expressed as FRC turnovers like LCI_2.5_.
